# Detecting glaucoma from multi-modal data using probabilistic deep learning

**DOI:** 10.3389/fmed.2022.923096

**Published:** 2022-09-29

**Authors:** Xiaoqin Huang, Jian Sun, Krati Gupta, Giovanni Montesano, David P. Crabb, David F. Garway-Heath, Paolo Brusini, Paolo Lanzetta, Francesco Oddone, Andrew Turpin, Allison M. McKendrick, Chris A. Johnson, Siamak Yousefi

**Affiliations:** ^1^Department of Ophthalmology, University of Tennessee Health Science Center, Memphis, TN, United States; ^2^German Center for Neurodegenerative Diseases (DZNE), Tübingen, Germany; ^3^ASST Santi Paolo e Carlo, University of Milan, Milan, Italy; ^4^Department of Optometry and Visual Sciences, City University of London, London, United Kingdom; ^5^NIHR Biomedical Research Centre, Moorfields Eye Hospital NHS Foundation Trust and UCL Institute of Ophthalmology, London, United Kingdom; ^6^Department of Ophthalmology, “Città di Udine” Health Center, Udine, Italy; ^7^Ophthalmology Unit, Department of Medical and Biological Sciences, University of Udine, Udine, Italy; ^8^IRCCS Fondazione Bietti, Rome, Italy; ^9^School of Computing and Information System, University of Melbourne, Melbourne, VIC, Australia; ^10^Department of Optometry and Vision Sciences, University of Melbourne, Melbourne, VIC, Australia; ^11^Department of Ophthalmology and Visual Sciences, University of Iowa Hospitals and Clinics, Iowa City, IA, United States; ^12^Department of Genetics, Genomics, and Informatics, University of Tennessee Health Science Center, Memphis, TN, United States

**Keywords:** deep learning, artificial intelligence, glaucoma, fundus photograph, visual field, automated diagnosis

## Abstract

**Objective:**

To assess the accuracy of probabilistic deep learning models to discriminate normal eyes and eyes with glaucoma from fundus photographs and visual fields.

**Design:**

Algorithm development for discriminating normal and glaucoma eyes using data from multicenter, cross-sectional, case-control study.

**Subjects and participants:**

Fundus photograph and visual field data from 1,655 eyes of 929 normal and glaucoma subjects to develop and test deep learning models and an independent group of 196 eyes of 98 normal and glaucoma patients to validate deep learning models.

**Main outcome measures:**

Accuracy and area under the receiver-operating characteristic curve (AUC).

**Methods:**

Fundus photographs and OCT images were carefully examined by clinicians to identify glaucomatous optic neuropathy (GON). When GON was detected by the reader, the finding was further evaluated by another clinician. Three probabilistic deep convolutional neural network (CNN) models were developed using 1,655 fundus photographs, 1,655 visual fields, and 1,655 pairs of fundus photographs and visual fields collected from Compass instruments. Deep learning models were trained and tested using 80% of fundus photographs and visual fields for training set and 20% of the data for testing set. Models were further validated using an independent validation dataset. The performance of the probabilistic deep learning model was compared with that of the corresponding deterministic CNN model.

**Results:**

The AUC of the deep learning model in detecting glaucoma from fundus photographs, visual fields, and combined modalities using development dataset were 0.90 (95% confidence interval: 0.89–0.92), 0.89 (0.88–0.91), and 0.94 (0.92–0.96), respectively. The AUC of the deep learning model in detecting glaucoma from fundus photographs, visual fields, and both modalities using the independent validation dataset were 0.94 (0.92–0.95), 0.98 (0.98–0.99), and 0.98 (0.98–0.99), respectively. The AUC of the deep learning model in detecting glaucoma from fundus photographs, visual fields, and both modalities using an early glaucoma subset were 0.90 (0.88,0.91), 0.74 (0.73,0.75), 0.91 (0.89,0.93), respectively. Eyes that were misclassified had significantly higher uncertainty in likelihood of diagnosis compared to eyes that were classified correctly. The uncertainty level of the correctly classified eyes is much lower in the combined model compared to the model based on visual fields only. The AUCs of the deterministic CNN model using fundus images, visual field, and combined modalities based on the development dataset were 0.87 (0.85,0.90), 0.88 (0.84,0.91), and 0.91 (0.89,0.94), and the AUCs based on the independent validation dataset were 0.91 (0.89,0.93), 0.97 (0.95,0.99), and 0.97 (0.96,0.99), respectively, while the AUCs based on an early glaucoma subset were 0.88 (0.86,0.91), 0.75 (0.73,0.77), and 0.92 (0.89,0.95), respectively.

**Conclusion and relevance:**

Probabilistic deep learning models can detect glaucoma from multi-modal data with high accuracy. Our findings suggest that models based on combined visual field and fundus photograph modalities detects glaucoma with higher accuracy. While probabilistic and deterministic CNN models provided similar performance, probabilistic models generate certainty level of the outcome thus providing another level of confidence in decision making.

## Introduction

Glaucoma is a heterogeneous group of disorders that represents the second leading cause of blindness overall, affecting up to 91 million individuals worldwide (1, 2). Since glaucoma can be asymptomatic in early and even intermediate stages, its diagnosis is often made only after irreversible damage of the optic nerve and loss of vision have already occurred (3). Hence, methods for predicting glaucoma could have a significant impact on public health.

Dilated fundus photography provides convenient and inexpensive means for recording optic nerve head (ONH) structure and glaucomatous optic neuropathy (GON) assessment remains a gold standard for indicating the presence of glaucoma (4, 5). However, manual assessment of the optic disc through fundus photographs for glaucoma screening requires significant clinical training, is highly subjective with currently limited agreement regarding results even among glaucoma specialists, and is labor intensive for application to the general population (6, 7). Moreover, there is a large variation in disk size among different populations and even within normal population making clinical diagnosis challenging (8, 9).

Standard automated perimetry (SAP) is a psychophysical test in which localized light stimuli are presented at pre-determined locations from fixation in random order to produce a map of local retinal sensitivity (10, 11). Currently, glaucoma is most often diagnosed by a combination of clinical examinations including evaluation of optic disc and visual field (12, 13). However, detecting glaucoma using visual fields remains challenging because visual field testing is subject-dependent, time-consuming, and visual fields are highly variable particularly in more deteriorated test points (14, 15).

Nevertheless, fundus photographs and visual fields have their limitations and benefits in glaucoma assessment and each testing modality may partially capture an aspect of this complex condition and be one piece of the glaucoma puzzle. We hypothesize that multiple modalities together may provide a better overall portrait of glaucoma and enhance its prediction. Models based on artificial intelligence (AI) may better suit multi-modal data analysis compared to conventional statistical models. Learning from data has advantages over predefined assumptions and rules to build the knowledge in machine learning classifiers. Recent advances in AI and deep learning models, along with significant growth in the amount of available data, have shown promise and allowed the development of objective systems for glaucoma diagnosis (16–21).

Deep learning models, however, require large clinically annotated training datasets to learn promising features from the images (22). Several studies have shown that deep learning models can identify disease-induced signs to diagnose disease or identify the severity of disease from ophthalmic images with high accuracy in ocular conditions such as diabetic retinopathy, age-related macular degeneration, and glaucoma (16–21, 23–27). If successful, deep learning models based on fundus photographs may provide automated assessment of glaucoma and impact population-based screening. Successful deep learning models based on visual fields, however, may improve glaucoma diagnosis and impact clinical practice.

Most of the applications of deep learning models, however, have been focused on using fundus photographs or other modalities in isolation to detect glaucoma, which may prove useful in screening applications. The aim of the current study is instead to assess the utility of a combination of fundus photographs and visual fields and comparing it to models that use either modality in isolation. Such models may be useful in clinical practice to identify glaucoma more accurately based on multiple data modalities. We hypothesize that combining structural and functional tests can improve detection rate compared to using a single modality.

Moreover, we partially address the black-box limitation of deep learning models by providing certainty level of the model on the generated outcome (how the model is confident in the likelihood of classification). Standard deep learning models are deterministic and do not account for the level of certainty (confidence) of the outcome (28). However, Bayesian neural network models could provide uncertainty by assigning prior distributions over network parameters (28). We thus propose a novel application of probabilistic Bayesian deep learning model that provide both likelihood and certainty of the outcome.

## Materials and methods

### Subjects

Subjects were recruited at eight study sites in Italy, United Kingdom, and US in 2017. Subjects were recruited in two separate studies in which the first study included 943 patients (dataset was used to develop and test the models) and the second study included 98 patients (dataset was used to independently validate models). Both eyes (one eye in the second study) of recruited subjects were tested. The fundus photographs and visual fields of this study were obtained from the Compass study coordinator. All patients had given their written informed consent to participate in the study in 2017. Ethics Committee and institutional review board (IRB) approvals were obtained (International Ethics Committee of Milan, Prot. N 0019459; clinical trial ISRCTN13800424). This study adhered to the tenets of the Declaration of Helsinki. The demographic information of the participants is provided in Table 1.

**TABLE 1:** Demographic characteristics of the study.

Characteristic	Development dataset	Independent dataset	Early glaucoma subset
Number of subjects	929	98	50
Number of eyes	1,655	196	86
Average age (years)	57.1	52.9	47.7
Average MD (dB)	–4.03	–4.06	–2.30
Average PSD (dB)	4.7	4.41	2.47
Race			
White	1,429 (86.3%)	144 (73.4%)	64 (74.4%)
Asian	51 (3.1%)	8 (4.1%)	5 (5.8%)
Black	24 (1.5%)	0	0
Unknown	116 (7.0%)	38 (19.4%)	11 (12.8%)
Other	35 (2.1%)	6 (3.1%)	6 (7.0%)

Details of inclusion and exclusion criteria can be found elsewhere (29). Briefly, the inclusion criterion for normal subjects was having a normal ONH in both eyes with no evidence of excavation, rim narrowing or notching, disc hemorrhages, or RNFL thinning and intraocular pressure (IOP) less than 21 mmHg in both eyes with no ocular pathologies, trauma, surgeries (apart from uncomplicated cataract surgery) in both eyes. Glaucoma eyes had GON defined as glaucomatous changes to the ONH or RNFL as determined by a specialist from fundus photograph or spectral-domain OCT, independently of the visual field, and an expert clinician confirmed the diagnosis of GON. Patients had to be receiving antiglaucoma therapy. Only patients with no ocular pathologies, trauma, surgeries (apart from uncomplicated cataract surgery) other than glaucoma in both eyes were included.

Standard assessment including axial length measurement with biometer, spectral-domain OCT of the ONH and RNFL, visual field examinations with HFA (Swedish interactive thresholding algorithm; SITA) and CMP (Zippy Estimation by Sequential Testing; ZEST), and fundus photo with CMP were performed.

The gold standard for annotating images was based on clinical diagnosis of GON from the clinical registry of the glaucoma clinics in the recruiting centers. An expert clinician confirmed the diagnosis of GON independently using the RNFL spectral-domain OCT or optic nerve photographs, independent of the visual field tests.

In an independent follow-up study and using a similar inclusion and exclusion criteria, 98 subjects were recruited for the purpose of validating the developed models.

### Fundus photographs

True color fundus photographs were collected using Compass (CMP, CenterVue, Padua, Italy) instruments. Compass is a fundus automated perimeter (FAP) and has been used in clinical practice recently (30). The structural dataset included 1,851 fundus photographs from normal subjects and patients with glaucoma in which 1,655 fundus photographs corresponded to the first study (development dataset) and were used to develop and test the models and 196 fundus photographs corresponded to the second study (validation dataset) and were used to independently validate the model.

### Visual fields

The functional dataset included 1,851 visual fields from normal subjects and patients with glaucoma in which 1,655 visual fields corresponded to development dataset and 196 visual fields corresponded to the validation dataset. Visual field examination was performed by CMP using a grid that contained all the 52 locations tested with HFA 24–2 but with only 1 blind spot location (instead of 2 as in the 24–2) and 12 additional points in the macular region of the VF using Zippy Estimation by Sequential Testing (ZEST) strategy. We excluded all 12 additional macular test points and used a grid which corresponded to HFA 24–2. As CMP is equipped with autofocusing, no near correction was needed. Visual field examinations were considered reliable if the false-positive frequency and the blind spot response frequency were less than 18 and 25%, respectively (29). If CMP visual field was deemed unreliable, the test was excluded from the analysis.

### Multi-modal dataset of paired fundus photographs and visual fields

As the fundus camera and VF testing systems are integrated in one Compass system, fundus photographs and VFs were collected at the same time. Eyes that both tests were reliable were included for the multi-modal analysis. The multi-modal dataset included 1,851 pairs of reliable fundus photographs and visual fields in which 1,655 corresponded to the first dataset and 196 corresponded to the second dataset.

### Image and visual field preprocessing

As Compass instrument retakes fundus images if the quality is low, the amount of imaging artifacts was minimal. We performed Gaussian filtering to all fundus photographs to mitigate the discrepancy among images. The images were then cropped and resized to 380 × 380 × 3 for the downstream analysis.

### Deep learning models

We developed three different variational Bayesian deep learning models based on fundus photographs, visual fields, and combined fundus photographs and visual fields and assessed the accuracy of the models in detecting glaucoma from structural, functional, and combined structure-functional data. To compare models with conventional CNN models, we also developed three deterministic CNN models based on three types of the input data modality.

### Deep learning model for detecting glaucoma from fundus photographs

**FIGURE 1:**
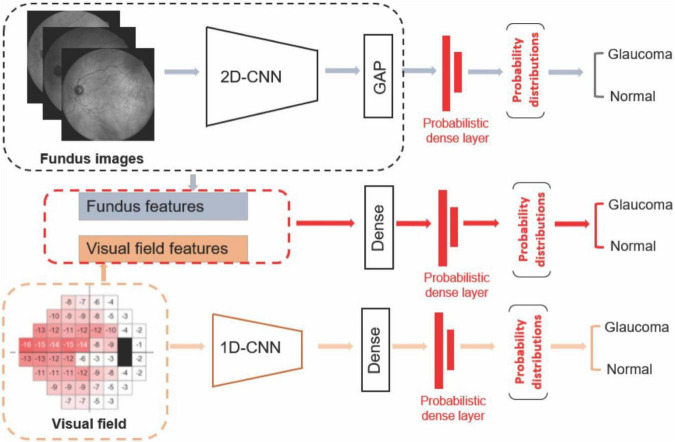
Diagram of the deep learning models. **Upper:** Deep learning model based on fundus photograph. **Middle:** Hybrid model based on combined visual fields and fundus photographs. **Lower:** 1-D CNN model based on visual fields.

The first deep learning model was developed using a probabilistic Bayesian convolutional neural network (CNN) based on fundus photographs (Figure 1). We employed the EfficientNetB4 (31) architecture and replaced the last fully connected layer of the CNN model with a global average pooling (GAP) layer followed by a probabilistic dense layer with two nodes representing two classes (glaucoma and normal).

To train the deep learning model, we used transfer learning. Transfer learning essentially transfers a common representation to the model and makes the convergence faster while providing more effective framework particularly when dealing with training datasets with small number of samples. A Gaussian distribution with standard deviation of 100 was applied on the parameters of the last dense layer as the priors, so the model training will be constrained by the Gaussian priors. The posterior distribution was approximated by a variational distribution and was estimated by minimizing the Kullback-Leibler (KL) divergence, or maximizing the evidence lower bound (ELBO) as Blundell et al. (28) and Loquercio et al. (32):

F(D,θ)=KL[q(W|θ)||P(w)]-E[logP(D|w)]q⁢(w|θ)

where *D* is data, ***w*** is network weights, and θ represent parameters. The cost function embodies a trade-off between satisfying the complexity of the data *D* and satisfying the simplicity prior *P*(**w**). Variational learning could identify parameters θ of the distribution on the weights *q*(**w**| θ) such that the KL is minimized (32). The model was forward propagated *T* times, and each time with a different parameter sampled from the posterior distribution. The output of the model is the mean and variance derived from *T* time predictions. Negative loglikelihood was used as a loss function with NAdam optimizer and initial learning rate of 0.0001. As we had a relatively large datasets, parameters of different layers, optimizers, and learning rates were very well fine-tuned during training.

The number of fundus photographs from normal subjects and patients with glaucoma were about similar thus provided a balanced dataset. We performed data augmentation on fundus photographs make the models independent of spatial transformations. We therefore applied random horizontal and vertical flips, rotations (0–45 degrees) and randomly changing the hue, saturation, and contrast. Batch sizes of 32 images were generated and NAdam optimizer were used for training.

### Deep learning model for detecting glaucoma from visual fields

The second deep learning model was developed using a probabilistic Bayesian 1-D CNN model based on visual fields (Figure 1). As there are only 52 visual field test locations present, we used a variational Bayesian 1-D CNN strategy to account for low-resolution aspect of visual field data and avoiding overfitting. Deep learning models have been previously used to analyze VF data as well (33). The input to the model were total deviation values at 52 visual field test locations. To train the 1-D CNN model for detecting glaucoma from visual fields, we used 512 filters with 5 kernels for the first layer, 512 filters and 3 kernels for the second layer, followed by a dense layer of 1,024 neurons with dropout of 0.25 and a final probabilistic dense layer. We used SGD optimizer at a learning rate of 0.0001 with momentum of 0.9 and decay of 0.0001 to train the visual field model.

### Deep learning model for detecting glaucoma from both fundus photographs and visual fields

The third AI model was a hybrid deep learning construct. More specifically, we first extracted the fundus features from the efficientnetB4 base model and GAP layers. We then concatenated deep fundus features with visual fields total deviation values followed by a dense layer composed of 512 neurons. We then added a probabilistic dense layer and used NAdam optimizer with initial learning rate of 0.0001 to train the hybrid model. All programs were implemented in Python with a backend of TensorFlow.

### Train, test, and validation

We had access to two independent datasets in the Compass study that had been collected separately. As such, we used the first dataset for development including training and testing (80/20 split) and used the second independent dataset to further re-test and independently validate the models. We therefore created three different datasets to develop and evaluate models: A full development dataset, a full independent validation dataset, and an early glaucoma subset (–4 dB < MD < –1 dB). The last subset provides a challenging scenario for the deep learning model as normal eyes and eyes with glaucoma were required to have MDs between –4 and –1 dB, thus harder to distinguish compared to full discovery and independent validation datasets.

We used fivefold cross-validation for training the models, hyperparameter selection, and testing. In each fold of the cross validation, the 80% of examples from each class were used for training while the remaining examples are used for testing. To avoid bias due to samples from both eyes of one subject in both training and testing subsets, we split the dataset based on subjects. We also repeated the development process five times each time selecting the training and testing (80/20 split) randomly to assure the model is not biased to sample selection.

### Visualization to partially illuminate deep learning black box

To identify the imaging markers for glaucoma diagnosis, we developed gradient-weighted class activation maps (GradCAMs) (34). We used two of the last convolutional layers of the CNN to produce a coarse localization map (34) of the regions of fundus photographs that were more important for the deep learning model to make diagnosis. Activation maps can validate the clinical relevance of the regions that derived the model and may also serve as imaging biomarkers for glaucoma.

### Statistical analyses and evaluations

We used fivefold cross-validation and computed the mean accuracy across all fivefold to mitigate data selection bias in training and testing. As we split dataset by subject ID, we compared the age and mean deviation (MD) of the datasets using *t*-test. We evaluated the accuracy of each model using area under the receiver operating characteristic curve (AUC) and compared the AUC of different models based on the DeLong et al. (35) approach. The standard deviation of the probability distribution from the probabilistic model was used as the metric for evaluating the certainty (confidence) of the model in prediction. All statistical analyses were performed in Python.

## Results

About 1% of the fundus photographs in the Compass dataset had major artifacts (ONH or macula missing, significant light reflectance, or large regions being black/white) and were excluded from the study. The average age of the participants was 56.4 ± 17.7 (mean ± Standard Deviation) years. Mean deviation (MD) and age were significantly different between normal eyes and eyes with glaucoma (*p* < 0.05). Table 1 shows the demographic characteristics of the study.

The development dataset included 840 fundus photographs, 840 visual fields, and 840 pairs of fundus photographs and visual fields from normal eyes and 815 fundus photographs, 815 visual fields, and 815 pairs of fundus photographs and visual fields from eyes with glaucoma.

The validation dataset included 108 fundus photographs, 108 visual fields, and 108 pairs of fundus photographs and visual fields from normal eyes and 88 fundus photographs, 88 visual fields, and 88 pairs of fundus photographs and visual fields from eyes with glaucoma.

**FIGURE 2:**
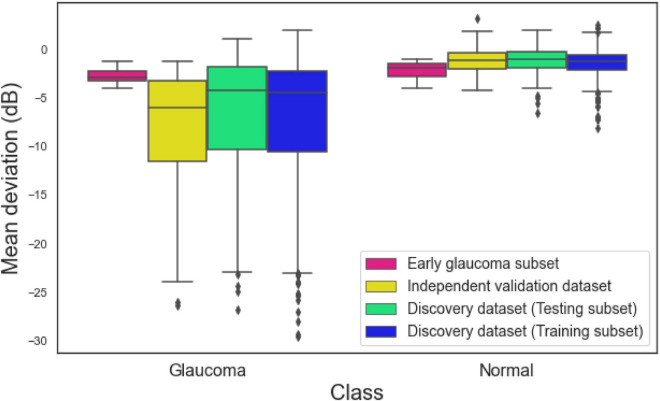
Mean deviation (MD) of eyes in the training subset of the discovery dataset, testing subset of the discovery dataset, independent validation dataset, and the early glaucoma subset.

Figure 2 illustrates the MD distribution of eyes in training and testing subsets of the development dataset, independent validation dataset, and an early glaucoma subset composed of eyes with –4 dB < MD < –1 dB. The eyes with glaucoma in the independent validation datasets had a worse MD compared to eyes with glaucoma in the development dataset. However, the MD of normal eyes and eyes with glaucoma in an early glaucoma subset were substantially closer compared to the distribution of MD of normal eyes and eyes with glaucoma in the full discovery or full independent validation datasets.

Table 2 presents numerous evaluation metrics (average values) of different probabilistic deep learning models in discriminating normal eyes from eyes with glaucoma. The AUC of the combined model based on full discovery, full independent validation, and early glaucoma subset were 0.94, 0.98, and 0.91, respectively, which was higher than the AUC of the single models. The accuracy of the combined model based on full discovery, full independent validation, and an early glaucoma subset were 0.85, 0.93, and 0.84, respectively, which was higher than the accuracy of the single models. Table 3 shows the details of the statistical difference between pairwise AUCs.

**TABLE 2:** Average value of evaluation metrics of different probabilistic deep learning models in discriminating normal eyes from eyes with glaucoma.

Dataset	Full discovery dataset			Full independent validation dataset			Early glaucoma subset		
Model	Fundus	Visual field	Combined	Fundus	Visual field	Combined	Fundus	Visual field	Combined
AUC	0.90 (0.89, 0.92)	0.89 (0.86, 0.91)	0.94 (0.91, 0.96)	0.94 (0.92, 0.95)	0.98 (0.98, 0.99)	0.98 (0.98, 0.99)	0.90 (0.88, 0.91)	0.74 (0.73, 0.75)	0.91 (0.89, 0.93)
Accuracy (%)	82 (79, 84)	81 (80, 82)	85 (82, 88)	88 (86, 89)	92 (91, 93)	93 (92, 95)	83 (80, 85)	68 (67, 69)	84 (82.86)
Sensitivity (%)	71 (67, 76)	0.73 (71, 76)	79 (72, 85)	77 (72, 82)	91 (89, 92)	91 (87, 95)	59 (48, 70)	100 (100, 100)	100 (100, 100)
Specificity (%)	94 (92, 96)	90 (88, 91)	92 (91, 93)	96 (95, 98)	94 (93, 95)	95 (94, 97)	95 (94, 96)	51 (50, 52)	75 (72, 78)

Numbers in parentheses reflect the 95% confidence intervals. AUC, Area under the receiver operating characteristic curve.

**TABLE 3:** Pair-wise comparison of the AUCs based on the method of Delong et al. (35).

Dataset	Model of AUC		*P*-value
Discovery dataset	Fundus	Visual field	0.081
	Fundus	Combined	0.063
	Visual field	Combined	0.000
Independent validation dataset	Fundus	Visual field	0.006
	Fundus	Combined	0.001
	Visual field	Combined	0.560
Early glaucoma subset	Fundus	Visual field	0.081
	Fundus	Combined	0.063
	Visual field	Combined	0.000

**FIGURE 3:**
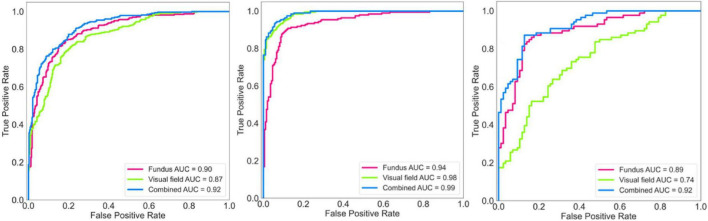
Receiver operating characteristic (ROC) curves of the deep learning model for diagnosing glaucoma. **Left:** ROC of the model for diagnosing glaucoma based on the discovery dataset. **Middle:** ROC of the model for diagnosing glaucoma based on the independent validation dataset. **Right:** ROC of the model for diagnosing glaucoma based on an early glaucoma subset.

Figure 3 (left panel) shows the ROC curves of the AI models applied on fundus photographs, visual fields, and combined modalities based on the discovery dataset. The AUC of the AI model for glaucoma diagnosis using combined modalities was 0.92 (0.91–0.96) which was higher than AUCs of 0.90 (0.89–0.92), 0.87 (0.86–0.91) for models based on fundus photographs and visual fields, respectively.

Figure 3 (middle panel) shows the ROC curves of the AI models applied on fundus photographs, visual fields, and combined modalities based on the independent validation dataset. The AUC of the AI model for glaucoma diagnosis using combined modalities was 0.99 (0.98–0.99) which was higher than AUCs of 0.94 (0.92–0.95), 0.98 (0.97–0.99) for models based on fundus photographs and visual fields, respectively.

Figure 3 (right panel) shows the ROC curves of the AI models applied on fundus photographs, visual fields, and combined modalities based on the early glaucoma subset (4 dB < MD < –1 dB). The AUC of the AI model for glaucoma diagnosis using combined modalities was 0.92 (0.89–0.93) which was higher than AUCs of 0.89 (0.88–0.91), 0.74 (0.73–0.75) for models based on fundus photographs and visual fields, respectively.

The specificity/sensitivity under the optimal threshold of the AI models based on fundus photographs, visual fields, and combined fundus/visual fields in the independent validation dataset were 90%/88%, 94%/89%, and 94%/100%, respectively. The sensitivity of the models based on fundus photographs, visual fields, and combined modalities at fixed 95% specificity were 71, 88, and 91%, respectively.

The AUC of the AI models for glaucoma diagnosis using combined fundus/visual fields data for detecting eyes at the early stages of glaucoma (MD ≥ –6 dB) was 0.98 (0.95–1.0) while the AUC of the model for detecting eyes at the later stages of glaucoma (MD < –6 dB) was 1.0 (0.99–1.0).

**FIGURE 4:**
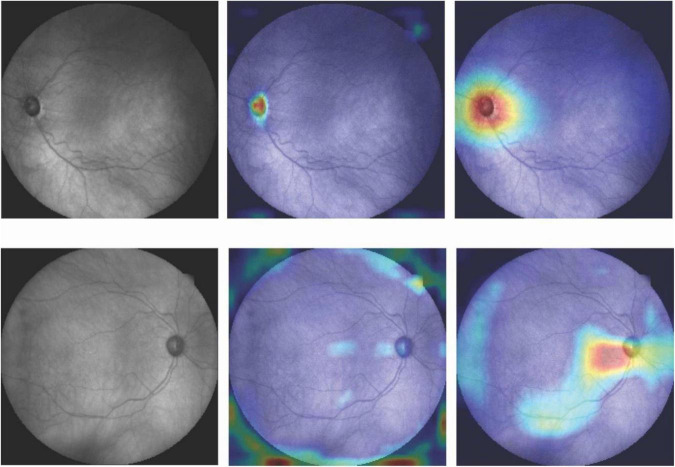
Fundus photographs and corresponding CAMs at two different convolutional layers. **Top row:** Correct prediction. **Bottom row:** Incorrect prediction. Highlighted regions were more important for the model to make diagnosis.

Figure 4 shows the activation maps of the deep learning model trained on fundus photographs. Two activation maps were derived from two different convolutional layers. Activation maps confirm that optic cup and rim areas were important regions for the deep learning model based on fundus photographs to make a diagnosis. Top row shows the fundus photograph and corresponding CAMs of an eye with glaucoma that was correctly classified as glaucoma. Bottom row shows the fundus photograph and corresponding CAMs of an eye with glaucoma that was incorrectly classified as normal. The model had focused on the optic disc region when the prediction was correct, while the focus was on the surrounding area of the optic disc when the prediction was incorrect.

**FIGURE 5:**
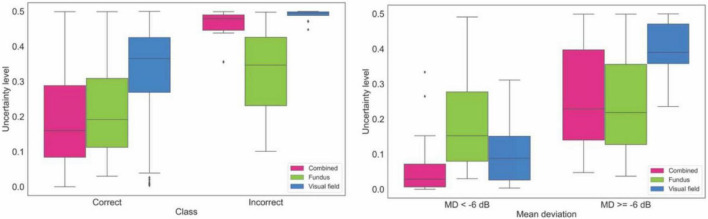
Uncertainty of the model in making diagnosis based on data from the independent validation dataset. **Left:** Uncertainty level of AI for making correct and incorrect diagnosis based on fundus photographs, visual fields, and combined modality. **Right:** Uncertainty level of AI in making diagnosis on fundus photographs, visual fields, and combined modality based on glaucoma severity levels.

Figure 5 (left panel) shows the box plots of the uncertainty of the model on making diagnosis based on fundus photographs, visual fields, and combined modality in the independent validation dataset. The uncertainty of the model in classifying eyes at two different VF severity levels is presented in Figure 5 (right panel). Based on the left panel, the AI model is more confident in making correct decision based on the combined modalities compared to AI models based on single modality. Additionally, AI models based on combined modalities and visual fields were highly uncertain when the diagnoses were incorrect.

Supplementary Figure 1 shows the MD distribution of eyes in the independent validation dataset that were classified correctly and incorrectly. Supplementary Figure 2 presents the ROC curves of the AI models for detecting glaucoma and normal eyes based on the independent validation dataset. Supplementary Figure 3 demonstrates the ROC curves of the AI models for detecting based on two severity level ranges of eyes in the independent validation dataset.

Supplementary Figure 4 shows the performance of the deterministic CNN models using fundus photographs, visual field, and combined fundus and visual field modalities based on the development dataset, independent validation dataset, and an early glaucoma subset. The AUCs of the CNN models based on the development dataset were 0.87 (0.85, 0.90), 0.88 (0.84, 0.91), and 0.91 (0.89, 0.94), respectively. Using the independent validation dataset, the AUCs were 0.91 (0.89, 0.93), 0.97 (0.95, 0.99), and 0.97 (0.96, 0.99), respectively, and the AUCs based on an glaucoma subset were 0.88 (0.86, 0.91), 0.75 (0.73, 0.77), and 0.92 (0.89, 0.95), respectively. Consistently, the model developed with combined modality provided a better performance compared to the models based on a single modality.

## Discussion

Unlike previous studies with the emphasis on deep learning models using a single imaging modality, we developed a hybrid deep learning model for detecting glaucoma from both structural and functional data. We found that the AUC of the probabilistic AI models based on combined modalities was consistently higher than AUC of the AI models based on a single modality. Additionally, the accuracy (and specificity/sensitivity) of the probabilistic AI model based on combined modality was consistently higher than the accuracy (specificity/sensitivity) of the AI models based on a single modality. This was more obvious when the eyes in the normal and glaucoma groups were more similar (analysis based on an early glaucoma subset with –4 dB < MD < –1 dB).

Fundus photography provides an affordable and portable means for documenting the status of the optic nerve. Since the invention of the early fundus cameras in early 1900’s, large datasets have been accumulated. As such, AI models have been applied extensively on fundus photographs for detecting glaucoma. Early AI models were mostly reliant on hand-engineering features (36, 37), however, with some user input, recent deep learning models can learn effective features in an unsupervised manner without requiring hand-engineering features or adopting ad hoc rules. Emerging deep learning models have now achieved varying AUCs in the range of 0.83–0.99 and accuracy in the range of 0.83–0.98.for screening and diagnosing glaucoma from fundus photographs (16–21). However, some of these studies have used relatively small datasets of fundus photographs, have not validated models using independent subsets, or have used fundus photographs mainly from eyes in the later stages of glaucoma, making generalization of findings challenging. Additionally, none of these studies have used multi-modal structural and functional data for glaucoma diagnosis. We, however, used two independent datasets that were collected over the course of two separate studies to develop and to independently validate the AI models. Both datasets included relatively similar number of fundus photographs from normal subjects and patients with glaucoma led to a balanced dataset contrary to most of the deep learning-based studies in the literature. This is critical as the AI models could simply become biased toward the class with majority number of samples.

Many studies have investigated the relationship between structure and function in glaucoma with the goal of improving glaucoma diagnosis and detection of glaucoma progression (38). Most of the previous studies made assumptions about the linearity of the structure-function relationship (39, 40), or other mathematical structure-function models (41). However, structural damage frequently correlates poorly with functional defects (42, 43). Artificial intelligence models, however, make no such structure-function assumptions and rather learn the relationships based on input structure and function data simultaneously. As such, these models may enhance glaucoma monitoring based on multi-modal data. When we compared the AUC of the models, the hybrid model (AI model based on combined modalities), however, performed better than models based on fundus photographs or visual fields using in independent validation subsets [*p* = 0.05; DeLong et al. (35) method]. The AUC of the hybrid model was also higher than the AUC of the AI model based on visual fields only. The difference was statistically significant for two of the datasets (Table 3, *p* < 0.01).

The average diagnostic AUC of the hybrid model based on the development dataset was 0.94 (95% confidence interval; CI: 0.91–0.96). To assure this AUC is not biased toward sample selection in training phase, we generated five different subsets by randomly selecting 80, and 20% of the development dataset for training and testing the hybrid model. We then compared the AUC of each model with the initial AUC and observed that the differences were not statistically significant (*p* > 0.4). Moreover, the MD distribution of the correctly and incorrectly classified eyes (Supplementary Figure 1) were similar for all AI models reflecting there was no bias. The diagnostic AUC of the hybrid model based on the independent validation dataset was 0.98 (95% CI: 0.98–0.99). This reflects a great degree of generalizability of the proposed hybrid model using unseen data from independent studies. However, the AUC of the hybrid model was greater (although not statistically different) than the model on development dataset which is not frequently observed. To explain this, we evaluated the glaucoma severity of the eyes in development and validation datasets. We observed that eyes with glaucoma in the independent validation dataset had a worse MD compared to glaucoma eyes in the development dataset (see Figure 2). As such, it was easier for the hybrid model to identify eyes at later stages of glaucoma compared to eyes in the development dataset.

The AUC of the hybrid AI models for glaucoma diagnosis using combined modalities based on the discovery dataset, independent validation dataset, and an early glaucoma subset were consistently higher than the AUC of the models based on a single modality (Figure 3 and Supplementary Figures 2, 3). To investigate the robustness of the AI model in dealing with more challenging datasets, we generated a new subset of normal eyes and eyes with glaucoma in which their MD was between –1 and –4 dB (considerable overlap between the severity level of normal eyes and eyes with glaucoma). The AUC of the hybrid AI model for glaucoma diagnosis was 0.92 while the AUC of the AI models based on fundus photographs and visual fields were 0.89 and 0.74, respectively. These finding suggests that the hybrid model utilizes the information from both modalities to make diagnosis and is robust in dealing with challenging datasets.

To further investigate the impact of glaucoma severity level on the performance of the three models, we further divided the eyes in the independent validation datasets to two severity levels: MD ≥−6 dB and MD < –6 dB, corresponding to mild-early and moderate-advanced stages of glaucoma, respectively. We then computed the AUC of the models based on eyes in these two groups. While the AUCs of the model based on fundus images, visual fields, and combined modalities for detecting eyes at the moderate-advanced stages of glaucoma were all 0.99 (0.97, 1.0), 1.0 (0.99, 1.0), and 1.0 (0.99, 1.0), the AUCs of the models based on fundus images, visual fields, and combined modalities for detecting eyes at the mild-early stages of glaucoma were 0.92 (0.90–0.94), 0.97 (0.95–0.99), and 0.98 (0.95–1.0), respectively. Higher AUC of the model in detecting eyes at the later stages of glaucoma compared to eyes at the earlier stages of glaucoma further validates the robustness and clinical relevance of the proposed model.

The diagnostic accuracy of the deep learning model based on fundus photographs proposed by Raghavendra et al. (16) was 0.98 and the diagnostic AUC of the model proposed by Li et al. (18) was 0.99. The lower AUC of our deep learning model based on fundus photographs could be due to several reasons. First, we used a highly balanced dataset with about equal number of fundus photographs from both groups in both development and validation datasets. However, the datasets used on those studies were not balanced where the AUC metric could be misleading. Moreover, about 50% of the eyes in our datasets were in the mild-early stages of glaucoma which is more challenging to detect compared to eyes at the later stages of glaucoma, which may have not been the case for those studies.

We also developed deterministic CNN models to compare with corresponding probabilistic CNN models. We observed that the performance of the probabilistic and deterministic models is quite similar (Figure 3, Supplementary Figure 4, and Supplementary Table 1). However, a major advantage of probabilistic CNN models compared to deterministic CNN models is providing both the likelihood and uncertainty/confidence level on the outcome. For instance, as expected, we observed (see Figure 5 right) that the proposed model is more certain in identifying eyes with glaucoma at the later stages of glaucoma (MD < –6 dB) compared to eyes at the earlier stages of glaucoma (MD ≥ –6 dB). Interestingly, the model was more certain (confident) in classification of those eyes that were classified correctly compared to eyes that were classified incorrectly. This is crucial for decision making systems considering the black-box nature of the CNN models. As a simple example, the model generates 95% likelihood for the input to belong to glaucoma and provide a 99% certainty (confidence in correct assignment) on the outcome which eventually provides the clinician more confidence level too.

Clinicians typically evaluate both visual field and ONH to diagnose glaucoma. Consistently, the CNN models based on combined modalities had higher confidence level in correct predictions compared to the CNN models that used a single modality. This corroborates the information theory concepts that combined modalities (structural and functional information) may provide complementary information regarding glaucoma thus improving the diagnosis.

Goldbaum et al. (44) used a mixture of Gaussian model for diagnosing glaucoma from visual field on a small dataset and obtained an AUC of 0.922, sensitivity of 0.79 at the specificity of 0.9. In another study, Bowd et al. (45) compared models developed based on structural (OCT) measurements, functional (visual field) measurements, and combined structural and functional measurements to diagnose glaucoma. They observed that the model employing combined modalities performed better compared with models that utilized a single modality, which is in agreement with our findings.

The Compass dataset that was used in our study had several strengths; participants were recruited from eight centers across the US and Europe, thereby reducing the idiosyncrasies of local datasets (29). Annotation was performed carefully based on both fundus photographs and OCT images by well-trained clinicians thus the amount of annotation biased is reduced compared to situations where only fundus photographs are used. Additionally, fundus photographs and visual fields were collected from same patients at the same time led to collecting structural and functional data under same condition with minimum time difference. However, it had several limitations too. One limitation was that the design of the Compass study only allows for a relative comparison of glaucoma detection while evaluation of how findings generalize to general population would require further validation based on additional independent datasets. Another limitation of the study is that only fundus photographs and visual fields were used to detect glaucoma while several other risk factors may contribute to glaucoma. Other limitation is related to risk of spectrum bias, particularly in our independent subset, that may affect the performance. We stratified eyes to early and moderate/advanced glaucoma severity levels and repeated the analysis to mitigate this limitation. We, however, were limited by the rather small validation subset and unable to stratify eyes to more severity levels to assess the impact of spectrum bias. Another source of bias is that glaucoma was defined by optic nerve appearance which may favor more the AI models based on fundus photographs. Therefore, future studies with datasets including other glaucoma risk factors are desirable to further investigate glaucoma diagnosis. It is worth mentioning that the eyes were collected from eye clinics and not general population thus the results may not support findings based on population-based studies.

## Conclusion

In conclusion, the hybrid probabilistic AI model based on combined modalities consistently provided higher accuracy compared to probabilistic AI models based on single modalities based on different datasets and selected challenging subsets. The proposed hybrid model also provides certainty (confidence) level on the outcome which adds another layer of confidence in diagnosis and may augment other routinely obtained medical examinations for glaucoma diagnosis. These methods may also identify previously unknown structural and functional signatures of glaucoma.

## Data availability statement

The original contributions presented in this study are included in the article/Supplementary material, further inquiries can be directed to the corresponding author/s.

## Author contributions

XH and JS designed the study, performed analyses, interpreted results, and drafted the manuscript. CJ provided clinic insight and explanation for the results of the model, arranged data collection, and transferring to our institute for this study. SY designed the study, performed analyses, justified analyses, drafted the manuscript, and acquired the data. All authors contributed to the article and approved the submitted version.

## Supplementary material

Supplementary Figure 1 Mean deviation (MD) the correct and incorrect classifications of the AI models from the independent validation dataset.

Supplementary Figure 2 Receiver operating characteristic (ROC) curves of the AI models for detecting glaucoma **(left)** and normal **(right)** eyes based on the independent validation dataset.

Supplementary Figure 3 Receiver operating characteristic (ROC) curves of the AI models for detecting glaucoma based on eyes in the independent validation dataset with mean deviations (MDs) of: **left:** MD ≥ –6 dB, **right:** MD < –6 dB.

Supplementary Figure 4 Receiver operating characteristic (ROC) curves of the deterministic CNN model for diagnosing glaucoma. **Left:** ROC of the model for diagnosing glaucoma based on the discovery dataset. **Middle:** ROC of the model for diagnosing glaucoma based on the independent validation dataset. **Right:** ROC of the model for diagnosing glaucoma based on an early glaucoma subset.
