# Supplementary material for: Detecting glaucoma from multi-modal data using probabilistic deep learning
Source: Front Med (Lausanne). 2022 Sep 29;9:923096. doi: 10.3389/fmed.2022.923096 (PMC9556968; doi:10.3389/fmed.2022.923096)
Supplement: Supplementary file 1 [file Table_1.docx]

| **Dataset** | **Deterministic Probabilistic** | | **p-value** |
| --- | --- | --- | --- |
| **Discovery dataset** | Fundus | Fundus | 0.056 |
|  | Visual Field | Visual Field | 0.206 |
|  | Combined | Combined | 0.529 |
| **Independent validation dataset** | Fundus | Fundus | 0.132 |
|  | Visual Field | Visual Field | 0.397 |
|  | Combined | Combined | 0.052 |
| **Early glaucoma subset** | Fundus | Fundus | 0.536 |
|  | Visual Field | Visual Field | 0.384 |
|  | Combined | Combined | 0.124 |

**Table s1.** Pair-wise comparison of the AUCs of deterministic and probabilistic model based on the method of Delong et al. ^35^.
