# Supplementary figures and images for: Detecting glaucoma from multi-modal data using probabilistic deep learning
Source: Front Med (Lausanne). 2022 Sep 29;9:923096. doi: 10.3389/fmed.2022.923096 (PMC9556968; doi:10.3389/fmed.2022.923096)

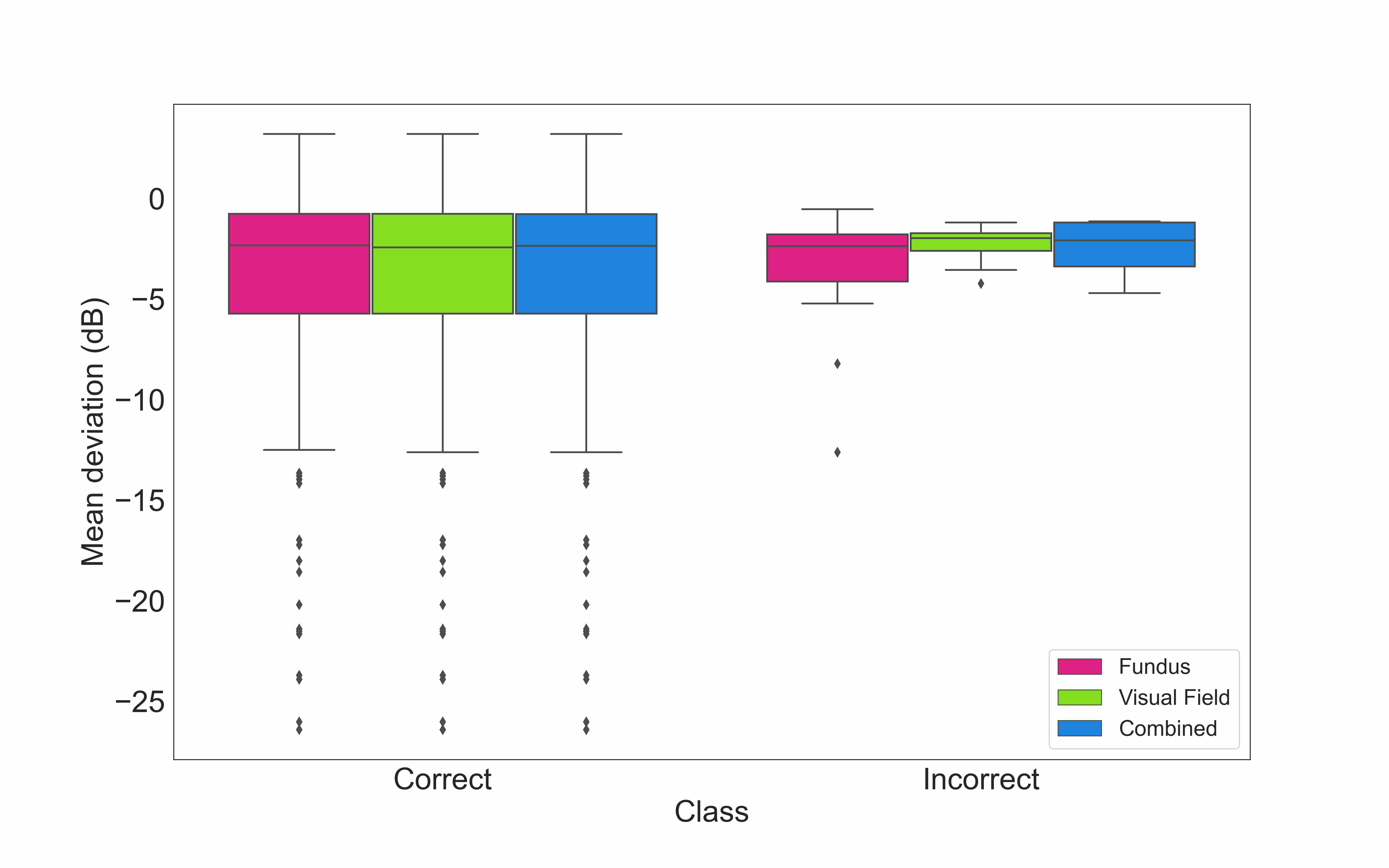

Supplement: Supplementary Figure 1 — Mean deviation (MD) the correct and incorrect classifications of the AI models from the independent validation dataset. [file Image_1.jpeg]

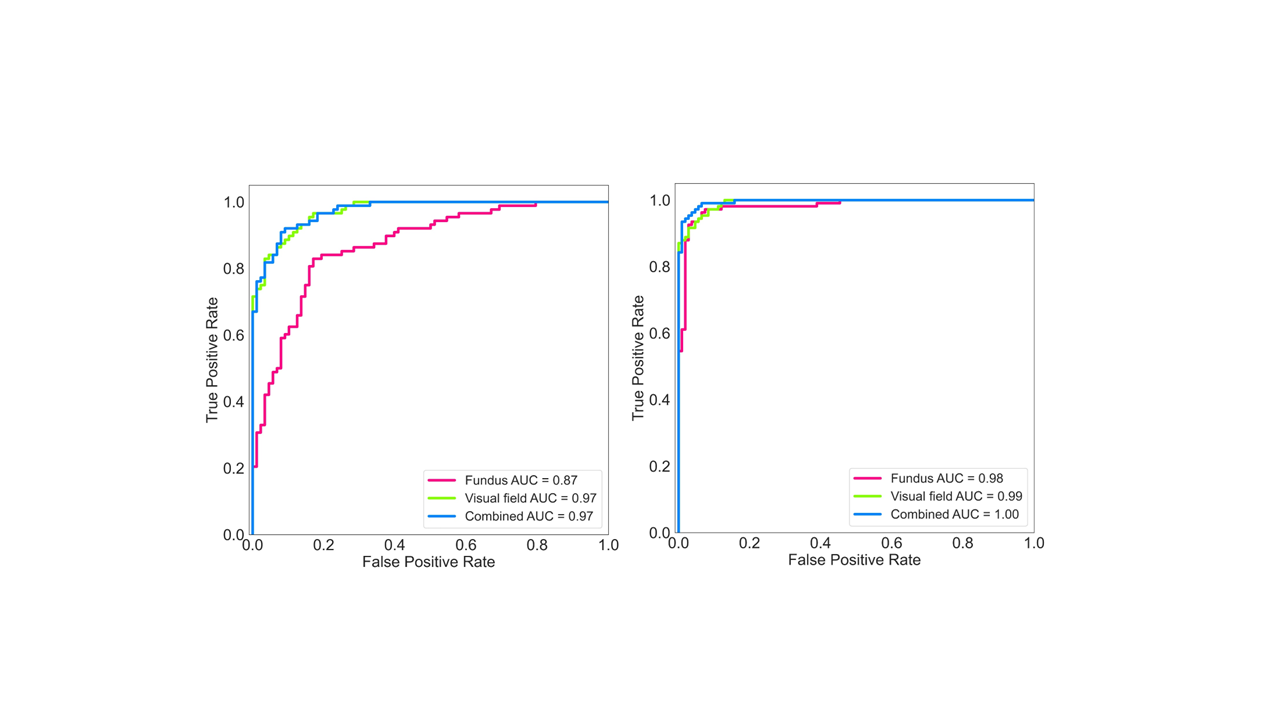

Supplement: Supplementary Figure 2 — Receiver operating characteristic (ROC) curves of the AI models for detecting glaucoma (left) and normal (right) eyes based on the independent validation dataset. [file Image_2.tif]

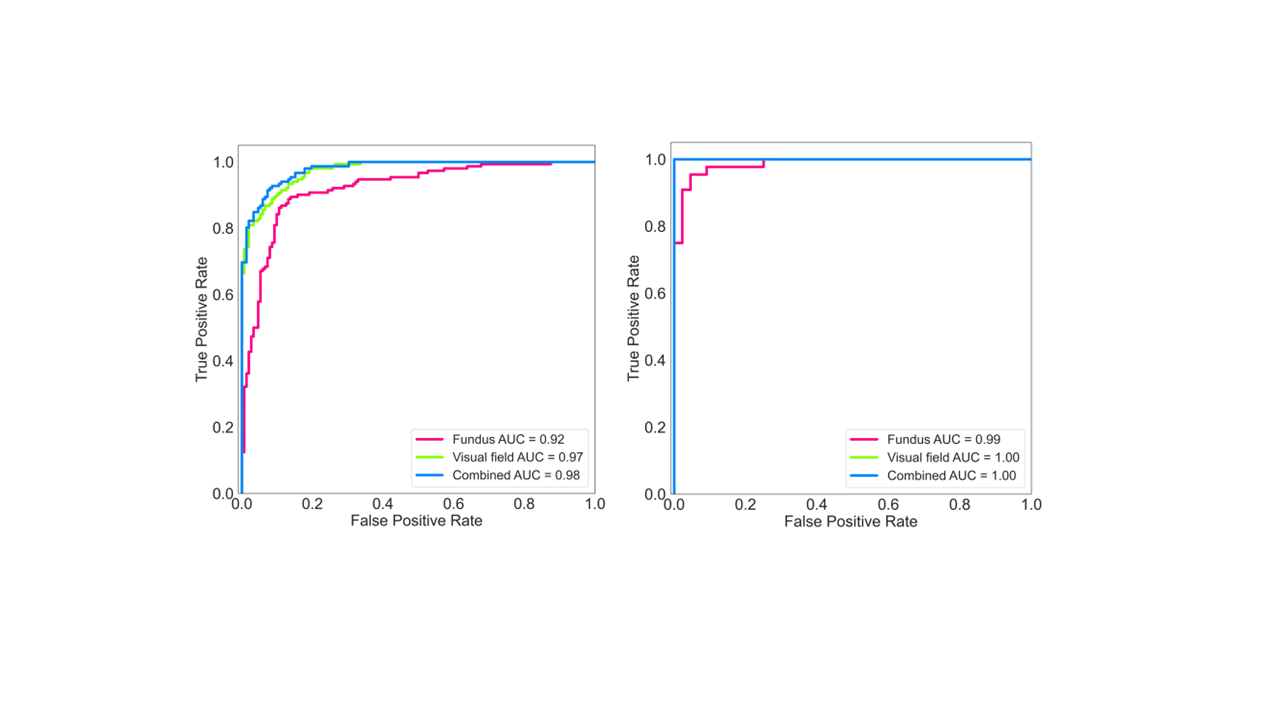

Supplement: Supplementary Figure 3 — Receiver operating characteristic (ROC) curves of the AI models for detecting glaucoma based on eyes in the independent validation dataset with mean deviations (MDs) of: left: MD ≥ –6 dB, right: MD < –6 dB. [file Image_3.tif]

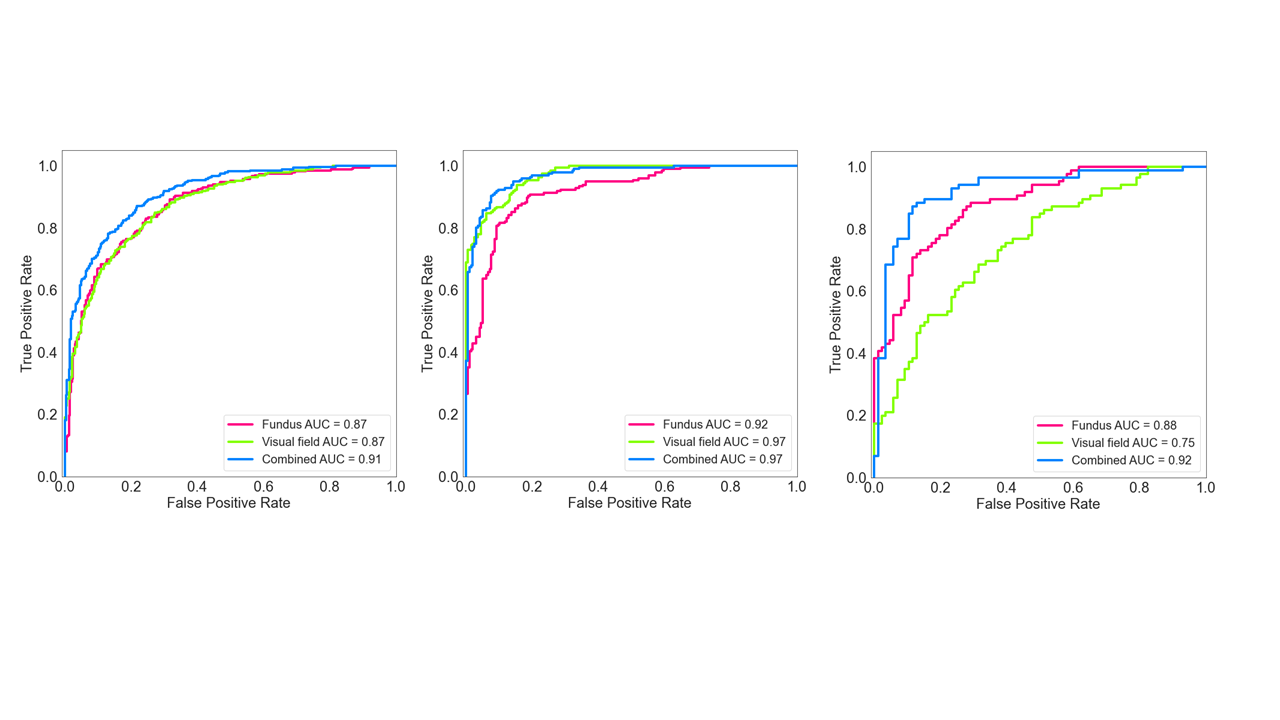

Supplement: Supplementary Figure 4 — Receiver operating characteristic (ROC) curves of the deterministic CNN model for diagnosing glaucoma. Left: ROC of the model for diagnosing glaucoma based on the discovery dataset. Middle: ROC of the model for diagnosing glaucoma based on the independent validation dataset. Right: ROC of the model for diagnosing glaucoma based on an early glaucoma subset. [file Image_4.tif]
